# Assessment of Pre-existing Type 2 Diabetes Mellitus Prevalence and Risk Factors Among Colorectal Cancer Patients in King Abdulaziz Medical City, Jeddah

**DOI:** 10.7759/cureus.32216

**Published:** 2022-12-05

**Authors:** Abdulaziz A Saber, Abdulrhman Alghamdi, Albara J Raggam, Loujen O Alamoudi, Alhanoof Aljahdali, Rayan M Bakheet, Abdulrahman M Algethmi, Muhammad A Khan, Syed Faisal Zaidi

**Affiliations:** 1 College of Medicine, King Saud Bin Abdulaziz University for Health Sciences, Jeddah, SAU; 2 Medical Education, King Saud Bin Abdulaziz University for Health Sciences, Jeddah, SAU; 3 Faculty of Eastern Medicine, Hamdard University, Islamabad, PAK

**Keywords:** kingdom of saudi arabia (ksa), body mass index (bmi), cigarette smoking, colorectal cancer, type 2 diabets mellitus

## Abstract

Background: Diabetes mellitus (DM) and cancer are recognized as non-communicable chronic disorders which are among the top ten causes of death globally. In Saudi Arabia, the prevalence of type 2 DM (T2DM) and colorectal cancer (CRC) is alarmingly high. Both T2DM and CRC share common risk factors. In this study, we aim to assess the prevalence of pre-existing T2DM among CRC Saudi patients.

Methods: In this cross-sectional study, data were collected from the medical records of 275 Saudi adult patients with CRC from 2009 to 2018 at King Abdulaziz Medical City, Jeddah (KAMC-J).

Results: Participants had a mean age of 57.0 years, standard deviation (SD) of 13.0, and were mostly males (60.00%) and Saudi (100.0%). Participants had a mean BMI of 26.42 (7.35) kg/m^2^. The prevalence of pre-existing T2DM in this study was 40.80%. 15.8% of participants were overweight and obese (BMI>30), respectively. The average age of diabetics and non-diabetics was 63.6 (10.64) and 52.73 (12.43), respectively. Diabetic patients are significantly older than non-diabetic patients (p<0.001). The average BMI for diabetics was 26.96 (7.26) kg/m^2^, whereas the average BMI for non-diabetics was 25.93 (7.48) kg/m^2^. No significant differences were found between the two groups.

Conclusion: This study provides new insight into the high prevalence of pre-existing T2DM in CRC patients in Saudi Arabia. In particular, the age of diagnosis of CRC in diabetic patients was significantly higher than in non-diabetics.

## Introduction

Diabetes mellitus (DM) and cancer are non-communicable chronic disorders that are among the top 10 causes of mortality worldwide [1]. DM is a metabolic disorder caused by either an insulin deficiency or insulin insensitivity [2]. In 2017, about 425 million adults worldwide had DM [3]. Colorectal cancer (CRC) is defined as a malignancy affecting the colon and rectal regions of the intestinal tract [4]. CRC is the third most common type of cancer, affecting 1.80 million people worldwide [5,6]. In Saudi Arabia, the prevalence of type 2 DM (T2DM) and CRC is alarmingly high [6]. The prevalence of T2DM in Saudi Arabia in 2015 is 32.8%, and the predicted prevalence in 2030 is 45.36% [7]. Additionally, CRC is the second most common type of cancer in Saudi Arabia [8]. Several studies have demonstrated that there are various common factors associated with T2DM and cancer, including CRC [9]. Furthermore, T2DM-related metabolic changes, such as hyperinsulinemia, hyperglycemia, and inflammation, have been implicated in carcinogenesis [9].

CRC and T2DM share common risk factors, such as diet, smoking, sedentary lifestyle, and obesity [10-12]. Diets based on large amounts of red and processed meat have a higher chance of causing CRC due to their carcinogenic properties [13]. Furthermore, diets high in carbohydrates may lead to elevated blood glucose levels, which is a risk factor for developing T2DM. A lack of physical activity has been reported as a risk factor for CRC and T2DM [14]. Specifically, a sedentary lifestyle may contribute to the development of obesity, which is a risk factor for CRC and T2DM. Individuals with a body mass index (BMI) greater than 25 kg/m^2^ and 30 kg/m^2^ are at a higher risk of developing T2DM and CRC, respectively [15]. Likewise, smoking is associated with T2DM development and CRC. Sattar et al. reported that smoking might increase the risk of T2DM by up to 1.5 times compared to non-smokers [16]. Moreover, in cigarette smokers, the incidence of CRC occurred at an earlier age, and 12% of CRC deaths were also associated with cigarette smoking [17].

It has been documented that exogenous insulin used for insulin-dependent diabetes elevates the risk of CRC via overstimulation of insulin-like growth factor receptor 1 (IGF-1R) in the intestinal epithelium, leading to proliferation and cell survival [9]. Likewise, hyperinsulinemia associated with T2DM is thought to have a similar effect, leading to CRC [17,18]. Additionally, chronic inflammation and decreased immunosurveillance associated with T2DM create a favorable environment for mutated intestinal epithelial cells to thrive and for CRC to develop [19,20]. 

Patients with co-existing diseases of DM and CRC have a higher risk of mortality than patients with CRC alone [9]. The morbidity and mortality rates of CRC are highly influenced by the anatomical location of the disease [21]. Patients with proximal CRC have an overall lower survival rate [21]. Studies addressing the association between T2DM and CRC location have found an increased risk of proximal and distal CRC in patients with T2DM [21]. Rectal cancer studies have also demonstrated an association. However, the data are inconsistent regarding gender-specific associations [21]. Furthermore, T2DM is associated with worse disease-free survival, with a chance of CRC recurrence in T2DM CRC survivors [9]. 

The link between T2DM and CRC has been reported in several studies [22,23]. However, the relationship between T2DM and CRC remains unclear [24]. Larsson et al., conducting a meta-analysis on the association between T2DM and CRC, found that T2DM was associated with an increased risk of CRC [22]. Likewise, Peeters et al. found a 1.3-fold increased risk of CRC in patients with pre-existing T2DM [23]. Globally, there is no association between T2DM and CRC incidence, which may suggest that differences between countries may affect the association between T2DM and CRC [24]. Data from some countries are insufficient [24]. Specifically, research assessing the link between T2DM and CRC in Saudi Arabia is lacking [24]. Furthermore, the association between T2DM and CRC is influenced by confounders such as diet, inflammatory bowel disease, alcohol consumption, and smoking [24]. Thus, in this study, we aimed to assess the prevalence of pre-existing T2DM in CRC Saudi patients. 

## Materials and methods

Study design, area, and setting

The data collection setting was the Medical Records Services Department at King Abdulaziz Medical City, Jeddah (KAMC-J). It was a cross-sectional study based on patient medical records accessed from the BEST CARE electronic health information system, as well as hard copy patient records. 

Identification of study participants 

The inclusion criteria for the study were all CRC patients with KAMC-J medical records between January 2009 and January 2018. Only adult patients of Saudi nationality were considered, defined by age 15 years or older. The exclusion criteria included any CRC patient with T2DM and other non-metastatic cancers. Additionally, CRC patients with unknown diabetes status were excluded.

There were a total of 305 documented CRC patients between 2009 and 2018 at KAMC-J. Based on the prevalence of pre-existing T2DM in CRC population in previous studies and using Raosoft® software (Raosoft Inc., Seattle, WA, USA), the estimated minimum required sample size was 146 for this study. However, to improve the accuracy of the prevalence estimation, all patients that met the inclusion criteria were considered subjects for this study. After applying the inclusion and exclusion criteria, a total of 275 subjects were recruited for this study.

Data collection process

The patients’ medical records were initially reviewed to ensure legibility for the study. Each patient's medical record was transferred by the data collectors to a Microsoft Excel spreadsheet, and a minimum of two data collectors double-checked each patient record to avoid any transcription errors. After sample selection, the prevalence of pre-existing T2DM in the CRC Saudi population was calculated.

Data analysis

All gathered parameters were coded into Microsoft Excel software and then transferred to JMP version 15.2.1 software (statistical analysis system [SAS] Institute Inc., Cary, NC, USA) for statistical analysis. Quantitative variables such as age, height, and weight were described as mean ± standard deviation. Qualitative variables were summarized using appropriate descriptive statistics, such as frequency, percentage, and bar graphs using GraphPad Prism 8 software. To compare quantitative variables, the independent t-test was used, and the Chi-squared test was used to compare qualitative variables. A p-value <0.05 was considered significant (95% confidence interval).

To maintain the confidentiality and anonymity of patient details, patient medical record numbers were collected and transferred to a separate coding sheet. Furthermore, information that may identify patients was not collected. In addition, all data, both hard and soft copies, were kept in a secure enclosure within the medical records premises accessible only to the research team. The data were stored in a password-protected file in Microsoft Office Excel. The study was approved by the Institutional Review Board (IRB) at King Abdullah International Medical Research Center (KAIMRC) with reference number: RYD-20-417780-62019.

## Results

At diagnosis of CRC, the diabetic and non-diabetic participants had a mean age of 57.10 years (SD 13.00), were mostly males (60.00%), and were Saudi (100.00%) (Table 1). Participants had a mean BMI of 26.42 (7.35) kg/m^2^ (Table 1), and most were married and unemployed (90.98% and 81.33%, respectively). The calculated prevalence of pre-existing T2DM in this study was 40.80% (Table 1).

**Table 1:** Study sample demographic data T2DM: Type 2 Diabetes Mellitus

Characteristics	Mean (SD)	N (%)
Age (at diagnosis)	57.10(13.00)	275(100.00%)
Body Max Index (kg/m^2^)	26.42(7.35)	274(100.00%)
Gender		
Male		165(60.00%)
Female		110(40.00%)
Ethnicity		
Saudi		275(100.00%)
Martial Status		
Single		9(3.38%)
Married		242(90.98%)
Divorced		1(0.38%)
Widowed		14(5.26%)
Job Status		
Employed		45(18.67%)
Unemployed		196(81.33%)
Pre-existing T2DM		
Yes		106(40.80%)
No		154(59.20%)

**Figure 1:**
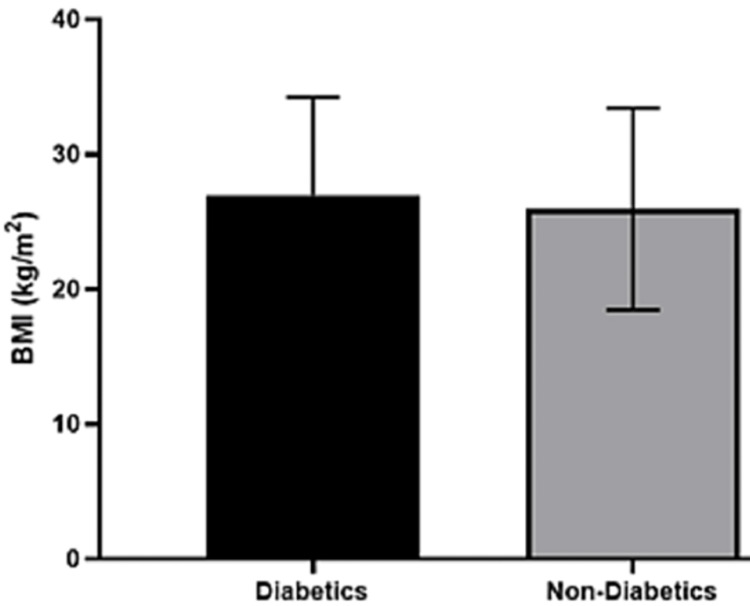
Comparing the body mass index of diabetics and non-diabetics colorectal cancer patients

The mean BMI for the diabetic group was 26.96 (7.27) kg/m^2^ in comparison to the non-diabetic group’s mean BMI of 25.93 (7.48) kg/m^2^. No significant difference was found between the two groups (Figure 1).

The BMI category containing the most participants was the normal BMI range, with 18.46% and 23.46% of the diabetic and non-diabetic groups, respectively (Table 2). In the diabetic group, 26.92% were males, while in the non-diabetic group, 33.46% were males (Table 2). 

**Table 2:** Diabetic and non-diabetic colorectal cancer patient demographic characteristics comparison

Characteristics	Diabetic N(%)	Non-Diabetic N(%)	Total N	P-value
Body Max Index (kg/m^2^)				
<18.5	3(1.15%)	14(5.38%)	260	0.681
18.5-24.9	48(18.46%)	61(23.46%)		
25.0-29.9	23(8.85%)	43(16.54%)		
30.0-34.9	32(12.31%)	36(13.85%)		
Gender				
Male	70(26.92%)	87(33.46%)	260	0.112
Female	36(13.85%)	67(25.77%)		
Saudi Province				
Makkah Region	68(27.76%)	115(46.93%)	245	0.002
Madinah Region	25(10.20%)	14(5.71%)		
Asir Region	2(0.82%)	8(3.27%)		
Al-Baha Region	5(2.04%)	2(0.82%)		
Jazan Region	0(0.00%)	3(1.22%)		
Hail Region	1(0.41%)	0(0.00%)		
Tabouk Region	0(0.00%)	2(0.82%)		
Living Status				
Alive	56(21.54%)	84(32.31%)	260	0.785
Dead	50(19.23%)	70(26.92%)		

Among the 13 provinces of Saudi Arabia, Makkah Province was the most common address for the participants (27.76% and 46.93% in the diabetic and non-diabetic groups, respectively, lived there), and the difference was found to be significant (p=0.002) (Table 2). Living status (alive five years after the CRC diagnosis) was found to be the highest in the non-diabetic group at 32.21% (Table 2).

Furthermore, only 3.65% and 6.25% of the diabetic and non-diabetic participants, respectively, had a positive family history of T2DM (Table 3). In addition, only 5.68% of the diabetic participants and 3.49% of the non-diabetic participants had a history of smoking (Table 3). 

**Table 3:** Colorectal Cancer Risk Factors In Diabetic and Non-Diabetic patients T2DM: Type 2 Diabetes Mellitus

Risk Factors	Diabetic N(%)	Non-Diabetic N(%)	Total N	P-value
Family History T2DM				
Positive	7(3.65%)	12(6.25%)	192	0.619
Negative	74(38.54%)	99(51.56%)		
Smoker				
Yes	13(5.68%)	8(3.49%)	229	0.057
No	84(36.68%)	124(54.15%)		

A total of 26.21% of the diabetic participants and 39.52% of the non-diabetic participants were initially diagnosed with stage 4 CRC (Table 4). The left colon was the most common CRC location reported-28.40% of the diabetic participants and 45.35% of the non-diabetic participants had CRC in the left colon (Table 4). Most patients in both groups had wild-type Kirsten rat sarcoma viral oncogene homolog (KRAS), neuroblastoma ras viral oncogene homolog (NRAS), and tumor protein 53 (Tp53) gene mutation (Table 4). 

**Table 4:** Colorectal Cancer Pathological Characteristics in Diabetic and Non-Diabetic patients CRC: Colorectal Cancer; KRAS: Kirsten rat sarcoma virus; NRAS: Neuroblastoma rat sarcoma virus; Tp53: Tumor protein p53

Colorectal Cancer	Diabetic N(%)	Non-Diabetic N(%)	Total N	P-value
CRC Stage				
Stage 1	3(1.21%)	4(1.61%)	248	0.988
Stage 2	13(5.24%)	17(6.85%)		
Stage 3	19(7.66%)	29(11.69%)		
Stage 4	65(26.21%)	98(39.52%)		
CRC Location				
Right-side	2(0.78%)	1(0.39%)	257	0.569
Left-side	73(28.40%)	116(45.14%)		0.251
Unspecified	30(0.38%)	35(13.62%)		0.381
Mutations				
KRAS			170	0.298
Mutant	16(9.41%)	37(21.76%)		
Wild-type	45(26.47%)	72(42.35%)		
NRAS			170	>0.99
Mutant	2(1.18%)	4(2.35%)		
Wild-type	59(34.71%)	105(61.76%)		
Tp53			169	>0.99
Mutant	1(0.59%)	2(1.18%)		
Wild-type	60(35.50%)	106(62.72%)		

**Figure 2:**
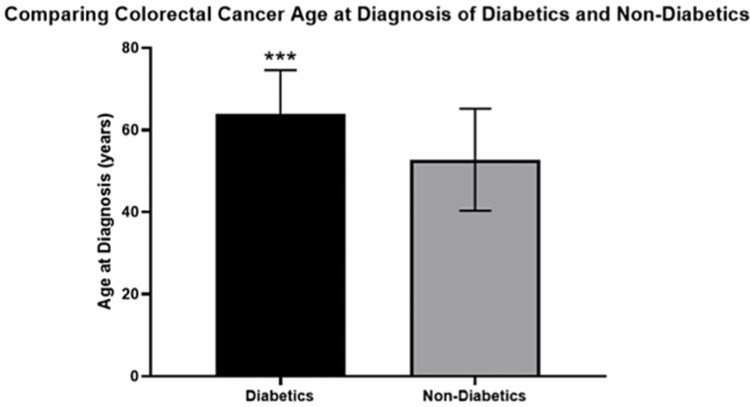
Comparing the age at diagnosis of colorectal cancer in diabetics and non-diabetics

In this study, the average age at diagnosis for the diabetic participants was 63.92 (10.64) years, and the average age at diagnosis for the non-diabetic participants was 52.73 (12.43) years (Figure 2). Hence, the diabetic participants were significantly older than the non-diabetic participants (p<0.001). 

**Figure 3:**
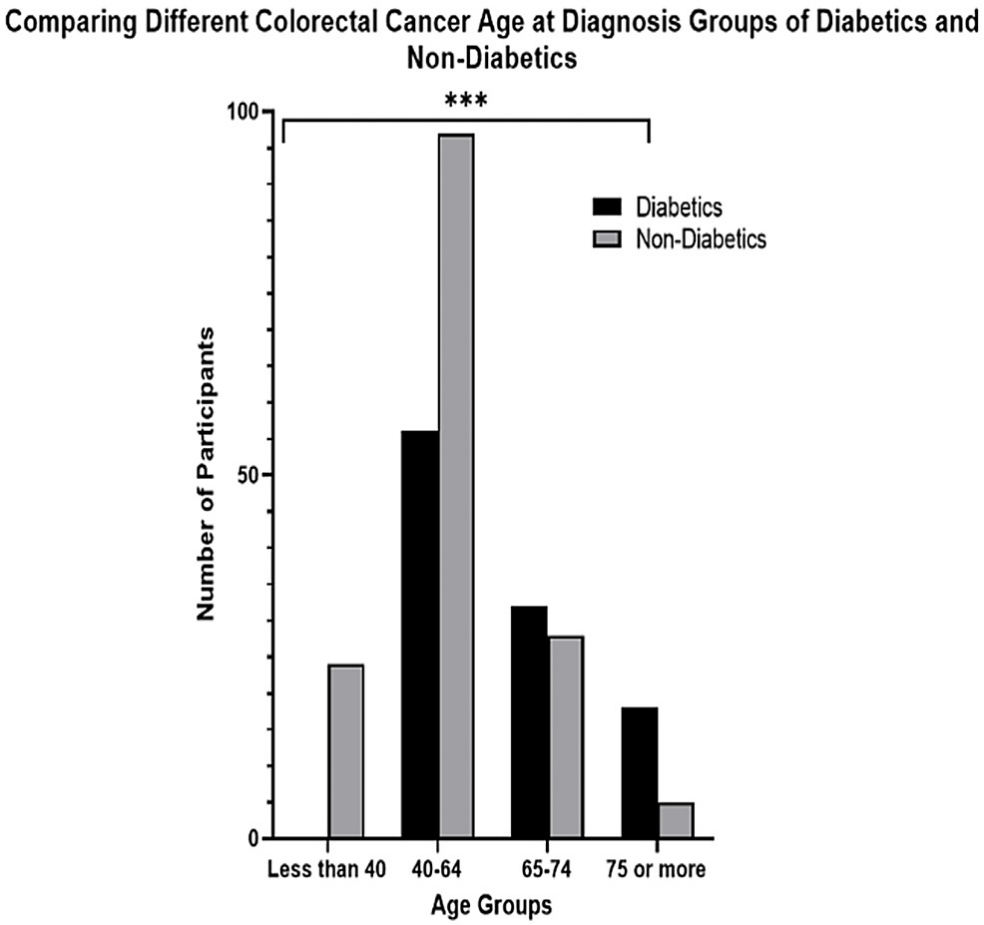
Comparing different age groups of diabetics and non-diabetics with colorectal cancer

A stratified analysis based on different age groups and DM status is depicted in Figure 3. In the group aged less than 40 years, no diabetic participants were found, whereas 24 in this age group were non-diabetic. In those aged 40-64 years, 56 were diabetic and 97 were non-diabetic, while in those aged 65-74 years, 32 were diabetic and 28 were non-diabetic. In the group aged 75 years or older, 18 were diabetic and 5 were non-diabetic. Therefore, for both the diabetic and non-diabetic participants, the highest numbers were found in the 40-64 years age group (Figure 3). The Chi-squared test showed a significant difference between the different age groups (p<0.001).

## Discussion

In this study, the prevalence of T2DM among Saudi patients with CRC was 40.8% (Table 1). This prevalence is higher than the previously reported prevalence of 23.6% in Taiwanese patients [25]. This discrepancy in the prevalence rate of T2DM among patients with CRC may be explained by region-specific factors, such as BMI, age, diet, and physical activity prevalence [26]. Moreover, the previous study had a sample size of 1,197 participants, which may explain the decreased prevalence [25].

It is mentioned in the literature that obesity increases the risk of colon cancer [27]. In this study, nearly half of the participants had a BMI graded as overweight or obese (Table 2). This follows the findings of Thygesen et al. who reported that a BMI above 22.5 notably increases the risk of colon cancer [28]. Furthermore, a five-unit increase in BMI has been shown to increase the risk of colon cancer in both genders based on a meta-analysis study [29]. However, the result was not significant between the diabetic and non-diabetic groups in this study, which could explain obesity as an independent risk factor for CRC. Also, a link between obesity, T2DM, and CRC is hypothesized based on molecular mechanisms and pathways but remains unclear in evidence-based medicine. Nevertheless, physical activity was not explored in this study. A sedentary lifestyle is a known risk factor for CRC and T2DM [14]. Moreover, previous studies reported a high prevalence of physical inactivity, overweight, and obesity in Saudi Arabia, which may contribute to the high prevalence rate of CRC and T2DM [30,31].

Furthermore, diabetic patients were significantly older than non-diabetic patients (Figure 1). Huang et al. also reported that diabetics were significantly older than non-diabetic patients [25]. The higher prevalence of T2DM in older CRC patients in this study (Figure 2) is consistent with the T2DM prevalence pattern in Saudi Arabia [32]. Al-Rubeaan et al. showed that the prevalence of T2DM in those aged below 35 years was 25.4%, but in those older than 45 years the prevalence was 40.2% [32]. According to the Saudi Cancer Registry, the CRC incidence rate increases between the ages of 40 and 75, which overlaps with the T2DM prevalence pattern [33]. This could explain the higher prevalence of patients with co-existing diseases in the older age group in this study. Thus, the association between age, T2DM, and CRC can be used to screen patients for early diagnosis of CRC.

Two large prospective cohort studies comprised a health study, which recruited 1,217,000 American female nurses, and a health professional’s follow-up study, which recruited 51,529 male healthcare workers [26,33]. The results after a 26-year follow-up showed that in male healthcare professionals, the hazard ratio (HR) of T2DM and the probability of CRC was 1.42 (95% CI: 1.21-1.81) [33]. Females had a weaker correlation between T2DM and CRC (HR = 1.17; 95% CI: 0.98-1.39) [26]. In the current study, it was found that there were also more diabetic males than diabetic females (Table 2). The distinctive HR of T2DM to CRC in men compared to women may be influenced by sex hormones, such as estrogen, which raise the responsiveness of peripheral target cells to insulin, and scientific research has shown an adverse correlation between estrogen and the occurrence of CRC [26].

Right-sided proximal colon cancer is more difficult to diagnose and has a worse prognosis than left-sided distal colon cancer, which could perhaps be explained by a different molecular tumor etiology [34]. One study found an increased risk of left-sided distant colon cancer among diabetics [21]. In this study, the majority of cases were diagnosed as left-sided distal colon cancer; however, there was no association between CRC location and T2DM. Furthermore, diabetic patients have been reported to have a higher TNM staging system when diagnosed with CRC [9], which was not seen in this study. Likewise, it has been hypothesized that patients with diabetes have a higher chance of developing a KRAS mutation; however, this study does not show a significant difference between the diabetic and non-diabetic groups [35]. The diversity of the results could be due to other environmental risk factors that are geographically specific and the complex multifactorial pathogenesis of the disease [24]. 

In cigarette smokers, T2DM, and CRC are more frequently associated, albeit slightly. In line with this observation, a demographic observational study found a higher correlation between the two diseases among smokers [36]. However, this study did not find any significance between smoking history, T2DM, and CRC (Table 3).

According to Khan et al., people with a genetic background of CRC are 2.4 times more likely to develop the disease before the age of 50 than people without a family history [37]. Likewise, those diagnosed with T2DM by the age of 50 had a nearly twofold greater chance of developing CRC than the general public [37]. Additionally, it was discovered that having both DM and a family medical history of CRC was linked to a nearly sevenfold increased risk of CRC compared to either condition separately [37]. The significant increase in CRC risk in the presence of DM and family medical history of CRC raises the possibility that these two variables interact, which calls for additional research, as this current study found no significance in the family history of T2DM [37].

A Saudi National Registry study found that most CRC cases in the Saudi population were found in the regions of Makkah, Riyadh, and the Eastern province [38]. This correlates with our study, which also found the highest number in the Makkah region (Table 2). A population-based study by Alyabsi et al. found a significantly increased risk of colon cancer in males in urban areas (relative risk [RR] = 1.14, 95% CI: 1.09-1.20). In addition, there was a higher risk for both genders for rectal cancer in urban areas (males: RR = 1.13, 95% CI: 1.06-1.19; females: RR = 1.13, 95% CI: 1.05-1.22) [38]. This difference between urban and rural areas may be explained by factors such as socioeconomic background, physical activity, and diet.

There are several limitations to this study. First, this study represents a retrospective cohort from a single institution with a small sample size. Since most expected patients are from the Makkah region, this would have resulted in Berkson’s bias. Second, other risk factors, such as physical activity and diet, were not assessed. Therefore, no possible association between risk factors, CRC, and T2DM was explored. Moreover, some medical record data reporting was inconsistent or missing. For example, T2DM diagnosis dates are often not reported.

## Conclusions

In conclusion, this study provides new insight into the high prevalence of pre-existing T2DM in CRC patients in Saudi Arabia, reaching 40.8%. In particular, the age at CRC diagnosis in diabetic patients was demonstrated to be significantly higher than in non-diabetic patients. Nearly half of the participants had a high BMI, which may contribute to the high prevalence of CRC, however, no significance was shown between diabetic and non-diabetic groups. No positive association was found between smoking and T2DM and CRC co-existence, nor family history of T2DM. Future studies should try to establish a causative relationship between the risk factors and consider exploring protective factors previously mentioned in the literature, such as the administration of metformin medication for this Saudi-based population.
